# Regulation of V-ATPase Assembly in Nutrient Sensing and Function of V-ATPases in Breast Cancer Metastasis

**DOI:** 10.3389/fphys.2018.00902

**Published:** 2018-07-13

**Authors:** Michael P. Collins, Michael Forgac

**Affiliations:** ^1^Program in Cell, Molecular and Developmental Biology, Sackler School of Graduate Biomedical Sciences, Tufts University, Boston, MA, United States; ^2^Department of Developmental, Molecular and Chemical Biology, School of Medicine, Tufts University, Boston, MA, United States

**Keywords:** V-ATPase, proton transport, acidification, regulated assembly, nutrient sensing, cancer, metastasis

## Abstract

V-ATPases are proton pumps that function to acidify intracellular compartments in all eukaryotic cells, and to transport protons across the plasma membrane of certain specialized cells. V-ATPases function in many normal and disease processes, including membrane traffic, protein degradation, pathogen entry, and cancer cell invasion. An important mechanism of regulating V-ATPase activity *in vivo* is regulated assembly, which is the reversible dissociation of the ATP-hydrolytic V_1_ domain from the proton-conducting V_0_ domain. Regulated assembly is highly conserved and occurs in response to various nutrient cues, suggesting that it plays an important role in cellular homeostasis. We have recently found that starvation of mammalian cells for either amino acids or glucose increases V-ATPase assembly on lysosomes, possibly to increase protein degradation (for amino acid homeostasis) or for the utilization of alternative energy sources (during glucose starvation). While regulation of assembly in response to amino acid starvation does not involve PI3K or mTORC1, glucose-regulated assembly involves both PI3K and AMPK. Another important form of V-ATPase regulation is the targeting of the enzyme to different cellular membranes, which is controlled by isoforms of subunit a. We have shown that V-ATPases are localized to the plasma membrane of highly invasive breast cancer cells, where they promote cell migration and invasion. Furthermore, overexpression of the a3 isoform is responsible for plasma membrane targeting of V-ATPases in breast tumor cells leading to their increased invasiveness.

## Introduction

### V-ATPase Function

Vacuolar H^+^-ATPases (V-ATPases) are ATP-dependent proton pumps present in intracellular membranes in all eukaryotes and at the plasma membrane of certain specialized cells (Forgac, 2007; Kane, 2012; Breton and Brown, 2013; Marshansky et al., 2014; Cotter et al., 2015b; McGuire et al., 2017). V-ATPase-dependent proton transport acidifies endocytic, secretory, autophagic and extracellular compartments, and is therefore essential for a variety of important processes (Forgac, 2007; Casey et al., 2010). Acidification of endosomes induces dissociation of internalized ligands from their cognate receptors, enabling receptor recycling and ligand targeting for degradation (Forgac, 2007; Maxfield, 2014). A similar process occurs in late endosomes, where acidification is required for dissociation of proteases from the mannose 6-phosphate receptor, facilitating enzyme delivery to the lysosome and receptor retrieval to the *trans*-Golgi network (Ghosh et al., 2003; Forgac, 2007). Furthermore, formation of endosomal carrier vesicles, which transport cargo between early and late endosomes, is also low pH-dependent (Gu and Gruenberg, 2000; Forgac, 2007). In synaptic vesicles, V-ATPases generate both a proton gradient and a membrane potential that drive neurotransmitter uptake (Farsi et al., 2016). V-ATPases also acidify secretory granules to facilitate the proteolytic processing of prohormones (Rhodes et al., 1987). Such processing also takes place in dendritic cell lysosomes, where internalized antigens are packaged for presentation on MHCII molecules (ten Broeke et al., 2013). Many pathogens co-opt endosomal acidification to gain entry into the cytoplasm, including diphtheria and anthrax toxin, as well as viruses such as influenza and Ebola (Gruenberg and van der Goot, 2006; Forgac, 2007; Grove and Marsh, 2011). Investigation into the role of V-ATPases in viral entry has provided valuable insight into the signaling pathways regulating V-ATPase activity in mammalian cells.

Acidification plays several essential roles in cellular nutrient and energy homeostasis. Amino acid homeostasis – in which cells balance amino acid generating and consuming processes – is dependent on V-ATPases, because lysosomal protein degradation is a major way that cells generate free amino acids (Xu and Ren, 2015). While the low pH of the lysosome is essential for proteolysis, the proton gradient imposed by the V-ATPase also drives the coupled export of amino acids into the cytoplasm (Xu and Ren, 2015). Another important energy-generating process is macroautophagy. During macroautophagy, cellular components are engulfed within double-membraned autophagosomes, which fuse with lysosomes to become autolysosomes (Feng et al., 2014). During this process, acidification is essential both autophagosome/lysosome fusion and for subsequent breakdown of intraluminal contents (Yamamoto et al., 1998; Kawai et al., 2007). In addition to maintaining the low pH of lysosomes, the V-ATPase associates with nutrient sensing machinery on the lysosomal surface and is required for recruitment of the metabolic regulators mTORC1 and AMPK (Zoncu et al., 2011; Zhang et al., 2014).

Finally, V-ATPases are localized to the plasma membrane of certain specialized mammalian cells where they function to transport protons from the cytosol to the extracellular space. Osteoclasts, epididymal clear cells, and renal alpha intercalated cells localize V-ATPases to the plasma membrane where they function in bone resorption, sperm maturation, and urine acidification, respectively (Breton et al., 1996; Frattini et al., 2000; Smith et al., 2000). Plasma membrane V-ATPases have also been observed in several cancer cell lines and human cancer samples. Our lab and others have obtained evidence that plasma membrane V-ATPases aid in cancer cell invasiveness and therefore represent an attractive therapeutic target to prevent metastatic spread in patients. This review will focus on recent contributions of our lab on regulated assembly of V-ATPases and their role in cancer cell invasion.

### V-ATPase Structure and Mechanism

**FIGURE 1:**
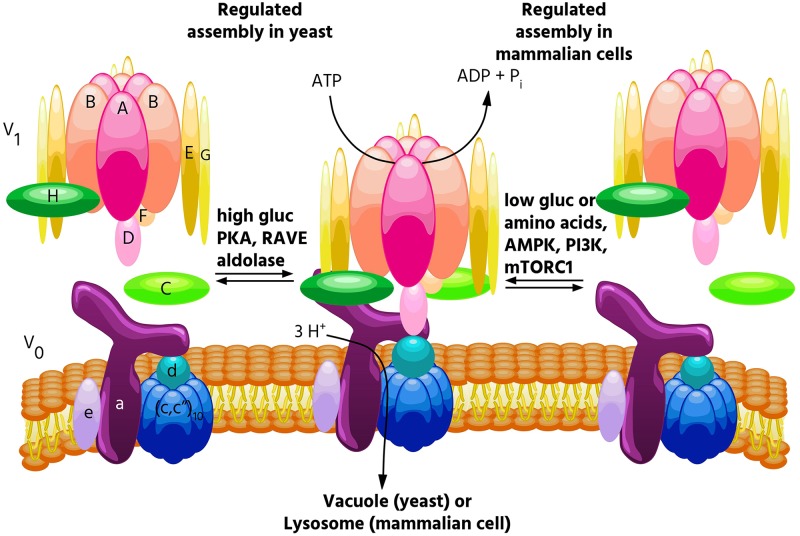
Regulated assembly of V-ATPases in response to nutrient availability. Reversible dissociation of the catalytic V_1_ and proton-conducting V_0_ domains is a major form of V-ATPase regulation and occurs in response to nutrient levels. In yeast, assembly increases when glucose is abundant (Kane, 1995), and this process is promoted by PKA (Bond and Forgac, 2008), the glycolytic enzymes aldolase (Lu et al., 2004) and phosphofructokinase (Chan and Parra, 2014), and the assembly factor RAVE (Smardon et al., 2002). By contrast, in mammalian cells, glucose starvation leads to increased V-ATPase assembly (McGuire and Forgac, 2018). This process is controlled by both the AMPK and PI3K/AKT pathways, but not PKA. Increased assembly in mammalian cells also occurs in response to amino acid starvation, and this effect is not controlled by PI3K or mTORC1 (Stransky and Forgac, 2015).

V-ATPases are large, multi-subunit complexes composed of a peripheral V_1_ domain that hydrolyzes ATP, and an integral V_0_ domain that translocates protons (Forgac, 2007; Zhao et al., 2015; Roh et al., 2018; **Figure 1**). V_1_ comprises eight distinct subunits designated A through H in a stoichiometry of A_3_B_3_CDE_3_FG_3_H, while V_0_ comprises five distinct subunits designated a, c, c″, d and e, in a stoichiometry of ac_9_c″de (yeast have an additional subunit, c′) (Cotter et al., 2015b). Subunits A and B are arranged in a hexameric configuration containing three catalytic ATP binding pockets (Forgac, 2007). ATP hydrolysis causes a conformational change in the hexamer which drives rotation of a central stalk composed of subunits D, F, and d, and a proteolipid ring composed of subunits c and c″ (Noji et al., 1997; Hirata et al., 2003; Forgac, 2007; Cotter et al., 2015b; Zhao et al., 2015; Roh et al., 2018). Each proteolipid subunit contains a buried glutamate residue that becomes protonated as the subunit rotates past the membrane-embedded C-terminal domain of subunit a (Forgac, 2007). Protons access the buried glutamate residues by entering from the cytoplasm through an aqueous hemichannel in subunit a (Forgac, 2007; Toei et al., 2011). These glutamate residues remain protonated as they are forced to rotate through the hydrophobic bilayer, and then deprotonate upon stabilization by a critical arginine residue in subunit a (Kawasaki-Nishi et al., 2001b). Protons then exit through a second hemichannel in subunit a, which faces either the lumen or extracellular space (Forgac, 2007; Toei et al., 2011). The AB hexamer resists the torque of rotation and is held stationary relative to subunit a by three EG heterodimers, which link the top of V_1_ to subunits C, H and the N-terminal domain of subunit a (Cotter et al., 2015b; Roh et al., 2018).

### V-ATPase Regulation

Because so many essential processes are pH-dependent, regulation of V-ATPase activity is tightly controlled. While several modes of V-ATPase regulation have been observed, among the most important is regulated assembly, in which the V_1_ and V_0_ domains undergo reversible dissociation from each other. In the dissociated state, both the catalytic function of V_1_ and the proton transport function of V_0_ are inactive (Forgac, 2007; Kane, 2012). Regulated assembly occurs in response to various nutrient cues, and the next section of this review will focus on the role of regulated assembly in nutrient sensing.

Another important form of V-ATPase regulation is the targeting of V-ATPases to different cellular membranes, which is controlled by isoforms of subunit a. In yeast, subunit a is encoded by two genes, *VPH1* and *STV1*, with Vph1p-containing V-ATPases being targeted to the vacuole, while Stv1p-containing V-ATPases are targeted to the Golgi (Manolson et al., 1994; Kawasaki-Nishi et al., 2001a). Mammals have four isoforms of subunit a, with isoforms a1 and a2 being localized primarily intracellularly, whereas a3 and a4 are targeted to the plasma membrane of osteoclasts and renal intercalated cells, respectively (Frattini et al., 2000; Smith et al., 2000). Importantly, V-ATPases have also been observed at the plasma membrane of several types of cancer cells, and our lab has demonstrated that the a3 isoform is particularly important in promoting breast cancer cell invasiveness (Hinton et al., 2009; Capecci and Forgac, 2013; Cotter et al., 2015a, 2016). The final section of this review will cover recent developments from our lab on the role of a subunit isoforms in cancer cell migration and invasion.

## The Role of Regulated Assembly in Nutrient Sensing

### Regulated Assembly in Yeast

The first demonstrations of regulated assembly of the V-ATPase were in yeast and in tobacco hornworm midgut cells (Kane, 1995; Sumner et al., 1995). In insects, assembly decreases during times of reduced feeding (such as molting), while in yeast, assembly decreases during glucose starvation. Since disassembled V-ATPases are catalytically inactive, decreased assembly during times of nutrient depletion is thought to help preserve cellular ATP stores. In yeast, V-ATPase disassembly upon glucose withdrawal is rapid, reversible and does not require new protein synthesis (Kane, 1995). Starvation-induced disassembly requires a catalytically active V-ATPase, which suggests that a particular conformation may be required for dissociation, and also requires an intact microtubule network (Parra and Kane, 1998; MacLeod et al., 1999; Xu and Forgac, 2001). Reassembly requires the glycolytic enzymes aldolase and phosphofructokinase, and the heterotrimeric RAVE complex, which directly binds the V-ATPase in a glucose-dependent manner (Smardon et al., 2002; Lu et al., 2004; Chan and Parra, 2014). Interestingly, RAVE only promotes assembly of Vph1p-containing V-ATPases (Smardon et al., 2014), and only V-ATPases localized to the vacuole undergo disassembly in response to glucose withdrawal, while V-ATPases localized to the Golgi do not (Kawasaki-Nishi et al., 2001c). Our laboratory has shown that regulated assembly of the yeast V-ATPase in response to changes in glucose availability is controlled by the Ras/cAMP/PKA pathway (Bond and Forgac, 2008). We identified Ira2p, a Ras GAP which is inhibited by elevated glucose, as a protein whose disruption leads to V-ATPase assembly even in the absence of glucose (Bond and Forgac, 2008). We further showed that expressing constitutively active Ras (Val19) or disrupting the regulatory subunit of PKA (both of which lead to constitutively active PKA) phenocopies the Ira2p deletion with respect to V-ATPase assembly (Bond and Forgac, 2008). Interestingly, V-ATPase assembly in yeast is sensitive to both extracellular and cytosolic pH (Dechant et al., 2010; Diakov and Kane, 2010). Furthermore, elevated cytosolic pH was found to promote PKA activity in a V-ATPase-dependent manner, suggesting a positive feedback loop between PKA and the V-ATPase may exist in yeast (Dechant et al., 2010). Yeast also increase V-ATPase assembly during salt and alkaline stress, and these assembly changes are controlled by levels of the signaling lipid PI(3,5)P_2_, while glucose-mediates effects are not (Li et al., 2014).

### Regulated Assembly in Mammals

V-ATPase assembly in mammalian cells is modulated by a diverse range of stimuli. As in yeast, mammalian V-ATPases respond to glucose availability. In several immortalized kidney cell lines, elevated glucose caused increased V-ATPase assembly and acidification of intracellular vesicles, perhaps to help maintain a neutral cytosolic pH during times of increased glycolysis (Sautin et al., 2005). It should be noted that these changes were observed at a glucose concentration of 25 mM, well above the normal physiological range of 3.5–5.5 mM. Whereas glucose-mediated assembly changes in yeast are controlled by PKA, increased assembly in response to elevated glucose was found to be PI3K-dependent in mammalian cells (Sautin et al., 2005). A major downstream effector of PI3K is the serine/threonine kinase mTORC1 (Lemmon and Schlessinger, 2010) (discussed below). Both PI3K and the Raf/MEK/Erk pathways were found to control V-ATPase assembly in cells infected with influenza virus (Marjuki et al., 2011). V-ATPase assembly also increases in dendritic cells during the process of maturation, where enhanced lysosomal acidification facilitates antigen processing (Trombetta, 2003). Our lab showed that inducing a semi-mature phenotype in primary cultured dendritic cells increases V-ATPase assembly and activity in a PI3K and mTORC1-dependent manner (Liberman et al., 2014). Finally, exposure of primary rat hepatocytes to epidermal growth factor (EGF) increased V-ATPase assembly, and this is thought to promote EGF-mediated mTORC1 activation by increasing cellular amino acid levels through enhanced proteolysis (Xu Y. et al., 2012). This study found that V-ATPase inhibition blocked EGF-stimulated mTORC1 activation but not activation of PI3K or Ras substrates (Xu Y. et al., 2012). Thus, the PI3K pathway has been implicated in most, but not all, instances of regulated assembly in mammalian cells.

Interestingly, the V-ATPase is required for the amino acid-dependent activation of mTORC1 (Zoncu et al., 2011). mTORC1 is a master metabolic regulator that integrates nutrient and growth factor levels to balance anabolic and catabolic processes. When adequate amino acids are present, mTORC1 is recruited to the lysosomal membrane where it contacts Rheb (Dibble and Manning, 2013). When activated by growth factors, Rheb activates mTORC1, which inhibits catabolic processes such as autophagy and promotes anabolic processes such as translation (Dibble and Manning, 2013). When V-ATPase function was disrupted either genetically or pharmacologically, mTORC1 remained inactive, even when amino acids were abundant (Zoncu et al., 2011). Because of this finding, and since reversible assembly is a major way that cells regulate V-ATPase activity, we wished to see if V-ATPase activity and assembly were modulated by amino acids. Indeed, we found that amino acids regulate the V-ATPase in mammalian cells, with amino acid starvation leading to a rapid and reversible increase in V-ATPase assembly and activity in lysosomes (Stransky and Forgac, 2015). Unlike other examples of regulated assembly in mammalian cells, this effect is PI3K and mTORC1-independent (Stransky and Forgac, 2015), indicating a novel signaling pathway regulates the V-ATPase in response to changes in amino acid availability. Although changes in V-ATPase assembly are not required for amino acid dependent changes in mTORC1 activity (Stransky and Forgac, 2015), we hypothesize that cells increase lysosomal V-ATPase assembly during amino acid starvation to enhance protein degradation, thereby increasing free amino acids. Thus, we propose that regulated assembly of the V-ATPase is a novel mechanism contributing to amino acid homeostasis.

In addition to being essential for mTORC1 activation by amino acids, the V-ATPase is also required for AMPK activation during glucose starvation (Zhang et al., 2014). AMPK directly senses and responds to cytosolic AMP/ATP ratios, such that during energy-poor conditions, AMPK inhibits anabolic processes (in part by inhibiting mTORC1), and promotes catabolic processes (Hardie, 2014). It was recently reported that AMPK, like mTORC1, is recruited to the lysosomal surface where it is activated, and that the V-ATPase is required for this process (Zhang et al., 2014). We have now shown that glucose starvation increases V-ATPase assembly and activity on lysosomes in mammalian cells (McGuire and Forgac, 2018). These effects are preceded by AMPK activation, indicating they are not required for the increase in AMPK activity upon glucose starvation (McGuire and Forgac, 2018). Furthermore, treatment of cells with the AMPK inhibitor dorsomorphin or the PI3K inhibitor LY294002 blocked the changes in V-ATPase assembly and activity, suggesting that these kinases regulate the V-ATPase in response to glucose starvation in mammalian cells (McGuire and Forgac, 2018). Future studies will focus on understanding the mechanism by which these signaling pathways modulate V-ATPase activity and testing the hypothesis that cells increase V-ATPase assembly during glucose starvation to promote the utilization of energy sources derived from autophagy.

## The Role of V-ATPases in Cancer Cell Invasiveness

V-ATPases are thought to aid in cancer cell survival through several mechanisms, including reducing cytosolic acid generated by aerobic glycolysis, conferring drug resistance through sequestration and efflux, and participating in oncogenic signaling pathways (for an extensive review see, Stransky et al., 2016). A number of studies have also proposed a role for plasma membrane V-ATPases in promoting cancer cell invasiveness, and this phenomenon has been best characterized in models of human breast cancer. Highly invasive MDA-MB-231 cells were found to possess greater plasma membrane V-ATPase activity than non-invasive MCF7 cells, and pharmacological V-ATPase inhibition reduced MDA-MB-231 cell migration and invasion *in vitro*, while having no effect on MCF7 cells (Sennoune, 2004). SKBR3 breast cancer cells also localize V-ATPases to the plasma membrane, and treatment with the V-ATPase inhibitor archazolid reduced their *in vitro* migration (Wiedmann et al., 2012).

Because cellular targeting of V-ATPases in normal cells is controlled by isoforms of subunit a, we hypothesized that a isoforms may be important for plasma membrane localization in breast cancer cells. We found that highly invasive MDA-MB-231 cells express 70-fold higher levels of a3 and 20-fold higher levels of a4 mRNA than non-invasive MCF7 cells (Hinton et al., 2009). Moreover, siRNA-mediated knockdown of either a3 or a4 reduced invasion to levels comparable to that observed with pan-V-ATPase inhibition by Concanamycin A (Hinton et al., 2009). This is a key result, because it suggests that targeted inhibition of specific isoform-containing V-ATPases can be as efficacious at blocking invasion as inhibiting all the V-ATPases in the cell. To compare more closely related cell lines, we next examined metastatic, Ras-transformed MCF10CA1a cells and the non-cancerous MCF10a breast epithelial line from which they were derived (Santner et al., 2001). MCF10CA1a cells express higher levels of a3 mRNA than MCF10a cells and localize more V-ATPases to the plasma membrane (Capecci and Forgac, 2013). siRNA-mediated knockdown of a3 or a3 plus a4 (but not a1 or a2) significantly inhibits invasiveness of MCF10CA1a cells (Capecci and Forgac, 2013). Furthermore, transient overexpression of a3 (but not the other a subunit isoforms) in MCF10a cells leads to increased plasma membrane V-ATPase localization and *in vitro* invasiveness (Capecci and Forgac, 2013). These results suggest that a3 specifically promotes invasiveness of breast cancer cells, in part by targeting V-ATPases to the plasma membrane. We next wished to test whether specific inhibition of cell surface V-ATPases could inhibit *in vitro* migration and invasion. We generated an MDA-MB-231 line stably expressing the c subunit with a C-terminal V5 epitope tag that is accessible to extracellular antibody binding when incorporated into plasma membrane V-ATPases. Treatment of cells with an anti-V5 antibody selectively inhibited plasma membrane V-ATPase activity in c-V5 expressing cells and inhibited both cell migration and invasion (Cotter et al., 2015a). The same effect was observed using a membrane impermeant form of the V-ATPase inhibitor bafilomycin (Cotter et al., 2015a). Both treatments reduced invasion to the same degree as pan-V-ATPase inhibition with a cell-permeant small molecule, suggesting that plasma membrane V-ATPases are essential to breast cancer cell invasiveness.

Utilizing isoform-specific antibodies, we have now confirmed that the invasive lines MDA-MB-231, SUM149 and MCF10CA1a localize more a3-containing V-ATPases to the plasma membrane than non-invasive MCF10a cells (Cotter et al., 2016). Moreover, when examining a cDNA array containing all stages of human breast cancer, we found a3 to be upregulated anywhere from 2.5 to nearly 50-fold in all 42 samples, relative to normal breast epithelial tissue (Cotter et al., 2016). a3 is localized to the leading edge of migrating breast tumor cells and is broadly upregulated in regions of invasive breast carcinoma relative to adjacent non-invasive tumors and normal tissue (Cotter et al., 2016). It should be noted that a3 has also been found to be important for metastasis of the B16 melanoma model (Nishisho et al., 2011). Taken together, these observations suggest that a3 containing V-ATPases at the plasma membrane of tumor cells may play an important role in cancer metastasis.

**FIGURE 2:**
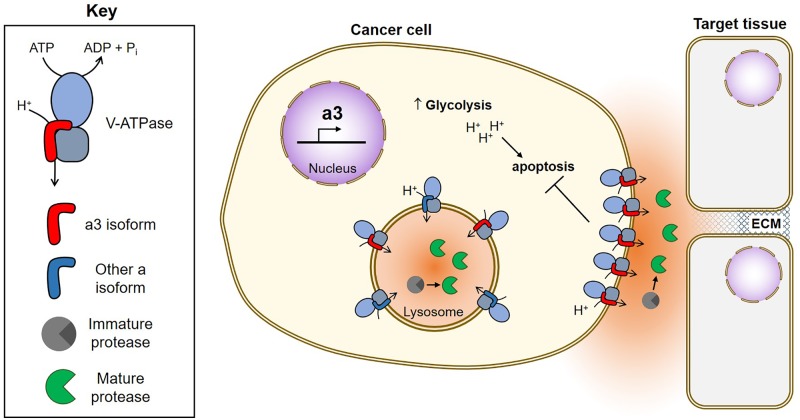
Function of V-ATPases in breast cancer survival and invasion. Breast cancer cells upregulate a3, which targets V-ATPases to the plasma membrane where they function in both cancer cell survival and invasion (Hinton et al., 2009; Capecci and Forgac, 2013; Cotter et al., 2015a, 2016). Plasma membrane V-ATPases promote cancer cell survival by removing acid generated from glycolysis (Sennoune, 2004; Xu J. et al., 2012; Avnet et al., 2013; von Schwarzenberg et al., 2013; Cotter et al., 2015a). Plasma membrane V-ATPases promote invasion by producing an acidic extracellular environment favorable to the function of cathepsins (Damaghi et al., 2013). Extracellular cathepsins may function in invasion by cleaving ECM components and/or by activating MMPs, which also function to degrade the ECM (Chung et al., 2011; Kulshrestha et al., 2015). Intracellularly, V-ATPases function in trafficking of acid-dependent proteases (not shown) and to promote protease maturation within lysosomes. Once activated, acid-dependent proteases are secreted by tumor cells to aid in invasion.

How do plasma membrane V-ATPases function in tumor cell invasion? One possibility is through the activation of secreted cathepsins, which can directly degrade components of the basement membrane and extracellular matrix, and also promote activation of other secreted proteases, such as matrix metalloproteases (Chung et al., 2011; Webb et al., 2011; Damaghi et al., 2013; Kubisch et al., 2014; Kulshrestha et al., 2015; **Figure 2**). Plasma membrane V-ATPases may create localized zones of low pH within partially sealed extracellular spaces that promote the activity of secreted proteases. Conversely, intracellular V-ATPases may also be important for invasion by activating proteases prior to their exocytosis. Nevertheless, it is highly encouraging that specific inhibition of plasma membrane V-ATPases is as efficacious as pan-V-ATPase inhibition at blocking invasion *in vitro* (Cotter et al., 2015a). This suggests it may be possible to specifically target cancer cells that overexpress plasma membrane and/or a3-containing V-ATPases to inhibit cancer cell invasion, while sparing most normal tissues. Targeted inhibition of a3-containing V-ATPases would have the added benefit of reducing osteoclast function, thereby inhibiting the ability of breast cancer cells to recruit osteoclasts for metastasis to bone (Steeg, 2016; Lambert et al., 2017). Future studies will be needed to determine whether these promising *in vitro* findings are confirmed using *in vivo* models of metastasis.

## Author Contributions

MC drafted the manuscript. MF revised the manuscript and approved the final version.

## Conflict of Interest Statement

The authors declare that the research was conducted in the absence of any commercial or financial relationships that could be construed as a potential conflict of interest.
